# 1,25-Dihydroxyvitamin D_3_-Conditioned CD11c+ Dendritic Cells are Effective Initiators of CNS Autoimmune Disease

**DOI:** 10.3389/fimmu.2015.00575

**Published:** 2015-11-18

**Authors:** Dario Besusso, Louise Saul, Melanie D. Leech, Richard A. O’Connor, Andrew S. MacDonald, Stephen M. Anderton, Richard J. Mellanby

**Affiliations:** ^1^MRC Centre for Inflammation Research, The University of Edinburgh, Edinburgh, UK; ^2^Centre for Multiple Sclerosis Research, The University of Edinburgh, Edinburgh, UK; ^3^Centre for Immunity, Infection and Evolution, The University of Edinburgh, Edinburgh, UK; ^4^Manchester Collaborative Centre for Inflammation Research, The University of Manchester, Manchester, UK; ^5^The Roslin Institute, Royal (Dick) School of Veterinary Studies, The University of Edinburgh, Midlothian, UK

**Keywords:** dendritic cell, vitamin D, experimental autoimmune encephalomyelitis, multiple sclerosis, T cell

## Abstract

Dendritic cells (DC) play a crucial role in regulating T cell activation. Due to their capacity to shape the immune response, tolerogenic DC have been used to treat autoimmune diseases. In this study, we examined whether 1,25 dihydroxyvitamin D_3_-conditioned bone marrow-derived DC (VitD-BMDC) were able to limit the development of autoimmune pathology in experimental autoimmune encephalomyelitis (EAE). We found that VitD-BMDC had lower expression of MHC class II and co-stimulatory molecules and were less effective at priming autoreactive T cells *in vitro*. Using our recently described BMDC-driven model of EAE, we demonstrated that VitD-BMDC had a significantly reduced ability to initiate EAE. We found that the impaired ability of VitD-BMDC to initiate EAE was not due to T cell tolerization. Instead, we discovered that the addition of 1,25(OH)_2_D_3_ to BMDC cultures resulted in a significant reduction in the proportion of CD11c+ cells. Purified CD11c+ VitD-BMDC were significantly less effective at priming T cells *in vitro* yet were similarly capable of initiating EAE as vehicle-treated CD11c+ BMDC. This study demonstrates that *in vitro* assays of DC function can be a poor predictor of *in vivo* behavior and that CD11c+ VitD-BMDC are highly effective initiators of an autopathogenic T cell response.

## Introduction

Dendritic cells (DC) are specialized sentinel cells that bridge the innate and adaptive immune response and play a crucial role in shaping the adaptive immune response (1). Depending on their activation status, DC can either activate or tolerize T cells. Activated DC upregulate co-stimulatory molecules and produce cytokines that drive T cell priming and effector differentiation (1). In the absence of activation, antigen presentation by steady-state DC can lead to T cell unresponsiveness and tolerance (1).

The ability of DC to tolerize T cells has resulted in the use of tolerogenic autologous DC as a treatment for autoimmune diseases (2). This approach is seen as highly attractive since it has the potential to limit the pathogenicity of autoreactive T cells without the need for widespread immunosuppression, which is a common side effect of many current therapies for autoimmune diseases (2, 3). A commonly used approach to generate tolerogenic DC involves the addition of immunomodulatory agents alongside GM-CSF to either monocyte or bone marrow cultures (2). The addition of the active vitamin D metabolite, 1,25 dihydroxyvitamin D_3_ (1,25(OH)_2_D_3_), to either murine bone marrow DC (BMDC) (4–6) or human monocyte-derived DC cultures (7–9), has been widely used to generate DC with a tolerogenic phenotype.

While the generation of clinical grade tolerogenic DC using 1,25(OH)_2_D_3_ has been shown to be feasible (7, 10, 11), the administration of autoantigen loaded DCs to patients with an ongoing autopathogenic T cell response is clearly not without risks. The safety profile of tolerogenic DC therapy has been difficult to assess in experimental models for a range of reasons, notably due to the difficulty of a robustly inducing autoimmunity through the passive transfer of DC. In experimental autoimmune encephalomyelitis (EAE), the widely used rodent model of multiple sclerosis (MS), few studies have demonstrated that the passive transfer of myelin-loaded DC can promote autoimmune pathology creating the impression that the transfer of DC in patients is unlikely to result in exacerbation of disease (12–14).

We have recently reported a novel model of BMDC-driven EAE in which mice develop a robust monophasic course of EAE following the transfer of traceable myelin basic protein (MBP)-reactive T cells and MBP-loaded, LPS-activated BMDC (15). In this study, we sought to understand whether 1,25(OH)_2_D_3_-conditioned BMDC (VitD-BMDC) were able to tolerize naïve T cells *in vivo*. We initially demonstrated that administration of 1,25(OH)_2_D_3_ completely protected mice from active EAE, whereas it afforded mice administered *ex vivo* activated T cells no protection, suggesting that vitamin D may play an important role in modulating the priming of naive T cells *in* vivo. We showed that BMDC generated in the presence of 1,25(OH)_2_D_3_ (VitD-BMDC) were significantly less effective at inducing EAE. Surprisingly, this was not due to a tolerizing effect on T cells with autopathogenic potential. Instead, we found that the addition of 1,25(OH)_2_D_3_ to bone marrow cultures resulted in a significant reduction in CD11c+ cells. Purified CD11c+ VitD-BMDC were significantly less effective at priming CD4+ T cells *in vitro* but were similarly effective at initiating EAE as vehicle-treated CD11c+ BMDC. This study demonstrates that CD11c+ VitD-BMDCs are in fact highly effective initiators of an autoaggressive T cell response *in vivo*, and highlights the fact that *in vitro* priming assays of DC function can be poor predictors of *in vivo* behavior and functionality.

## Materials and Methods

### Mice, Antigens, and Tissue Culture Medium

B10.PLxC56BL/6 and Tg4 CD45.1 mice were bred under specific pathogen-free conditions at the University of Edinburgh, and all experiments had local ethical approval and were performed in accordance with UK legislation. Tg4 mice express a transgenic T cell receptor (TCR) recognizing the Ac1-9 peptide of MBP in association with I-A^u^ (16). The MBP Ac1-9(4Lys) and a Ac1-9(4Tyr) analog peptide were obtained from Cambridge Research Biochemicals (Cleveland, UK). Tissue culture medium (RPMI 1640 medium) was supplemented with 2 mM l-glutamine, 100 U/ml penicillin, 100 μg/ml streptomycin, and 5 × 10^−5^ M 2-ME (all from Invitrogen Life Technologies, Paisley, UK) and 10% FCS (Sigma-Aldrich, Dorset, UK).

### Active Induction of EAE and 1,25(OH)_2_D_3_ Administration

B10.PLxC57BL/6 (CD45.2) mice received 2 × 10^6^ Tg4.CD45.1 CD4^+^ T cells. One day later (day 0), mice received 10 μg of the Ac1-9(4Tyr) peptide emulsified in CFA containing 50 μg of heat-killed *Mycobacterium tuberculosis* H37Ra (Sigma-Aldrich, Dorset, UK) at a total final volume of 100 μl injected s.c. into the hind legs. On the day of immunization and 48 h later, each mouse also received 200 ng of pertussis toxin (Health Protection Agency, Dorset, UK) in 0.5 ml PBS i.p. Clinical signs of EAE were assessed daily with the following scoring system: 0, no signs; 1, flaccid tail; 2, impaired righting reflex and/or gait; 3, partial hind limb paralysis; 4, total hind limb paralysis; 5, hind limb paralysis with partial front limb paralysis; 6, moribund or dead. Two hundred nanograms of 1,25(OH)_2_D_3_ (Sigma-Aldrich, Dorset, UK) in 200 μl soybean oil (Sigma-Aldrich, Dorset, UK), or vehicle alone, were administered i.p to mice every 48 h from day −1 to day 7 relative to day of immunization.

### Generation of Tg4 T Effector Cells and Passive Induction of EAE

Tg4.CD45.1 splenocytes were cultured at 4 × 10^6^ cells per ml with 10 μg/ml MBP(Ac1-9), 25 ng/ml rIL-12, 0.5 ng/ml rIL-2 (both from R and D systems), and 25 ng/ml rIL-18 (MBL, Nagoya) as described previously (17). Cells were harvested after 72 h culture and 3 × 10^6^ blasts were transferred i.v (day 0). On the day of cell transfer each mouse also received 200 ng of pertussis toxin (Health Protection Agency, Dorset, UK) in 0.5 ml PBS i.p. Clinical signs of EAE were assessed as described above. Administration of 1,25(OH)_2_D_3_ was also undertaken as described above.

### Generation of BMDC, Cytokine Analysis, and Primary Tg4 T Cell Activation Assays

Bone marrow dendritic cells were generated in the presence of recombinant GM-CSF (Peprotech, London, UK) for 9 days as previously described (18). Briefly, bone marrow was collected from tibias of B10.PLxC57BL/6 mice, and clusters within the bone marrow suspension were dispersed by vigorous pipetting. Cells were seeded into 6 well plates at 2 × 10^5^/ml in 2 ml 10% FCS medium with the addition of 20 ng/ml GM-CSF. At day 3, a further 2 ml of medium containing 20 ng/ml GM-CSF was added to each well. At days 6 and 8, 2 ml of culture supernatant was removed and replaced with 2 ml fresh culture medium containing 20 ng/ml GM-CSF. Vehicle or 1,25(OH)_2_D_3_ was added to the BMDC culture media at the concentration indicated in the text initially and at all subsequent media changes. To activate the BMDC, the cells were harvested at day 9 and were re-plated at 2 × 10^6^ BMDC/ml with 0.1 μg/ml lipopolysaccharide (LPS) (Sigma-Aldrich, Dorset UK) and 5 ng/ml of GM-CSF with 0.1 μg/ml Ac1-9(4Tyr) for an additional 18 h. In some experiments, CD11c+ BMDC were separated by FACS sorting prior to overnight stimulation with LPS and MBP. Cytokines were measured in BMDC supernatants by Flowcytomix simplexes as per manufacturer’s instructions (eBioscience, San Diego, CA, USA).

To study the primary activation of Tg4 T cells, varying numbers (as stated) of BMDC were cultured with 2 × 10^4^ CD4^+^ Tg4.CD45.1+ T cells per well (round bottomed, 96 well plates). The CD4+ T cells were purified using microbeads as per manufacturer’s instructions (Miltenyi Biotech, Surrey, UK). After 48 h, cell proliferation was assessed by the addition of [^3^H]thymidine (PerkinElmer, Cambridge, UK) at 0.5 μCi/well for the last 18 h of culture. [^3^H]Thymidine incorporation was measured using a scintillation β-counter (Wallac, Milton Keynes, UK). The results are expressed as mean counts per minute (c.p.m.) ± standard error of the mean (SEM) Tg4. T cell production of cytokines (IL-2, IL-10, IL-17, and IFN-γ) was assessed in culture supernatants by ELISA using paired monoclonal antibodies and recombinant cytokine standards purchased from BD Biosciences (NJ, USA). GM-CSF and TNF-α was detected using GM-CSF Ready-SET-Go ELISA (ebioscience, San Diego, CA, USA). IL-2 was measured in supernatants after 48 h of culture and IFN-γ, TNF-α, GM-CSF, IL-10, and IL-17 were measured after 72 h of culture.

### BMDC-Driven EAE

B10.PLxC57BL/6 mice received 2 × 10^6^ Tg4.CD45.1 CD4^+^ T cells. One day later, mice received 2 × 10^6^ LPS-stimulated, MBP Ac1-9(4Tyr) pulsed BMDC or CD11c + BMDC in a total volume of 100 μl injected s.c (50 μl into each hind leg). On the day of BMDC transfer, each mouse also received 200 ng of pertussis toxin (Health Protection Agency, Dorset, UK) in 0.5 ml PBS i.p. Clinical signs of EAE were assessed daily as described above.

### Preparation of Mononuclear Cells and FACS Analysis

Mice with EAE were sacrificed by CO_2_ asphyxiation, perfused with cold PBS and mononuclear cells were prepared from the brain and spinal cord as described previously (17). Single-cell suspensions were made from the spleen and draining lymph nodes, red blood cells were lysed using an ammonium chloride buffer (Sigma-Aldrich, Dorset, UK) and the cells were then resuspended in FACS buffer (PBS, 2% fetal calf serum, 0.01% sodium azide (Sigma-Aldrich, Dorset, UK)). Fc receptors were blocked with supernatant from the hybridoma 2.4G2. All antibodies were from eBioscience, Hatfield, UK, except where stated; LIVE/DEAD fixable cell stain (Life Technologies), anti-CD4-PerCP, anti-CD4-AF700 (BD Pharmingen, Oxford, UK), anti-CD11c-FITC, anti-Ki67-PE, anti-CD11b-efluor450, anti-CD45.1-FITC, anti-CD44-APC-Cy7, anti-CD80-PE, anti-CD86-APC, anti-CD62L-SA350, anti-Foxp3-PE, anti-GM-CSF-PE, Armenian hamster IgG-PE, and OX-6-FITC (AbD Serotec, Kidlington, UK). For intracellular staining in response to peptide, cells were resuspended at 1 × 10^7^/ml in the presence or absence of 20 μM 4Lys MBP. After overnight culture, 1 μl/ml of brefeldin A (e-bioscience, Hatfield, UK, 1000× stock) was added for the last 4 h of culture. Cells were surface stained prior to processing for intracellular staining using proprietary buffers according to the manufacturer’s instruction (e-bioscience for transcription factor staining or Becton Dickinson for cytokine staining). FACS data were collected using LSR Fortessa (BD Biosciences, NJ, USA) and analyzed using FlowJo software (Tree Star, Olten, Switzerland).

### Statistical Analysis

Statistical analysis of results was performed using the Mann–Whitney *U* test, the two-tailed Student’s *t*-test, and Fischer’s exact test as appropriate. Cytokine concentrations are presented as mean concentration ±SEM. Significance was set at *p* < 0.05.

## Results

### Administration of 1,25(OH)_2_D_3_ Is Protective Against Active but Not Passive EAE

**Figure 1:**
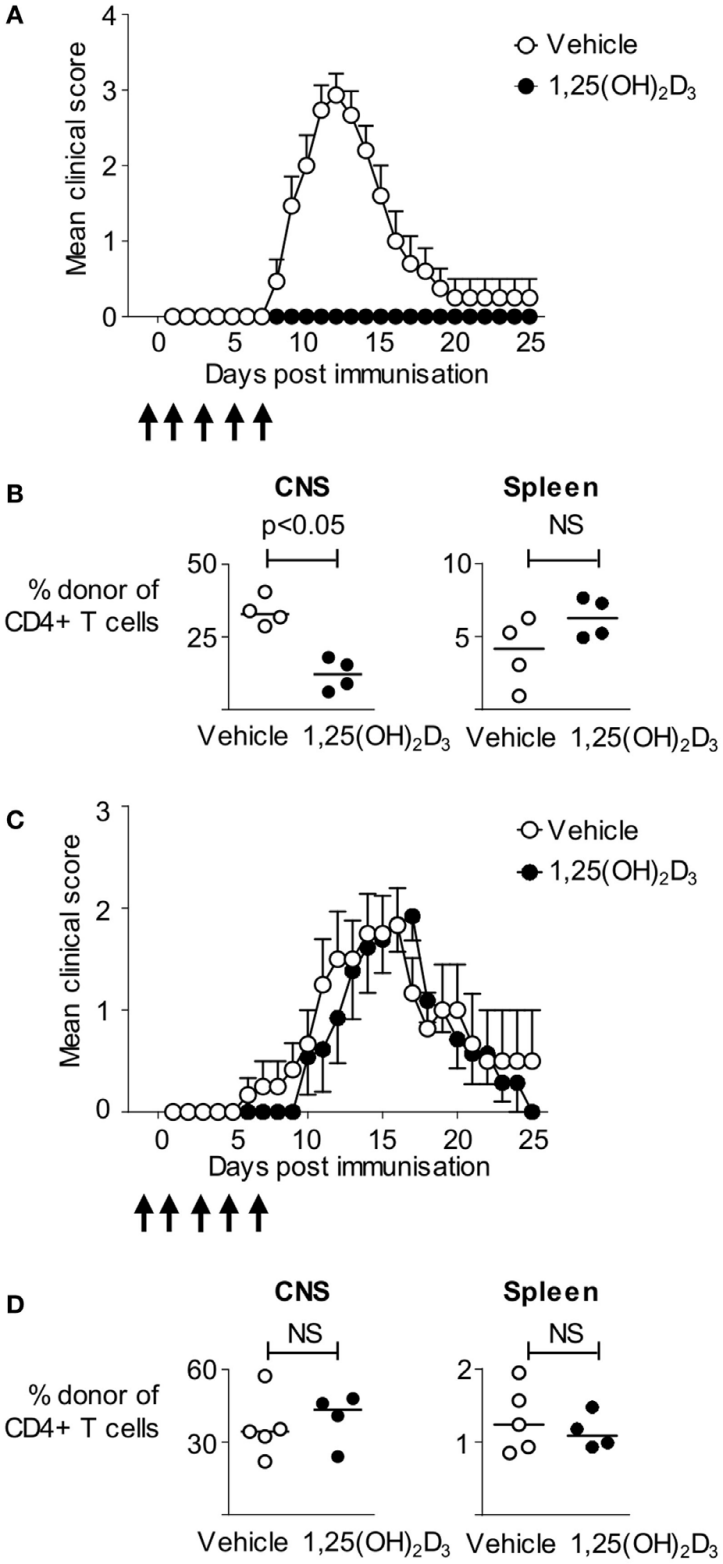
**Administration of 1,25(OH)_2_D_3_ is protective against active, but not passive, EAE**. B10.PLxC57BL/6 hosts were seeded with 2.5 × 10^6^ Tg4.CD45.1 CD4^+^ T cells prior to immunization for EAE induction with MBP peptide. 1,25(OH)_2_D_3_ (200 ng) (*n* = 14) or vehicle (*n* = 15) was administered as described in Section “Materials and Methods.” **(A)** Disease course. **(B)** Four mice in each group were sacrificed at day 13 for flow cytometric analysis of total CD4+ T cells, and donor CD4+CD45.1+ T cells. Cell percentages were compared by a Mann–Whitney *U* test. Results shown are pooled from two independent experiments. EAE was induced in B10.PL hosts by transfer of 3 × 10^6^ Tg4.CD45.1 effector T cells. Mice received 1,25(OH)_2_D_3_ (*n* = 13) or vehicle (*n* = 12) as described in Section “Materials and Methods.” **(C)** Clinical EAE course. **(D)** Five vehicle and four 1,25(OH)_2_D_3_-treated mice were sacrificed at day 18 post T cell transfer for flow cytometric analysis of total CD4+ T cells, and donor CD4+CD45.1+ T cells. Cell numbers and percentages were compared by a Mann–Whitney *U* test. Results shown are pooled from two independent experiments.

1,25(OH)_2_D_3_ has been shown to ameliorate pathology in a wide range of EAE models, most notably in mice immunized using CFA containing spinal cord homogenate (19), or immunodominant peptides of myelin oligodendrocyte glycoprotein (MOG), or MBP (20–30). Here, we made use of an informative T cell transfer model in which non-transgenic host mice are first seeded with naïve CD4^+^ T cells from Tg4 mice expressing a transgenic TCR recognizing the Ac1-9 peptide of MBP, prior to immunization with the Ac1-9(4Tyr) MBP peptide. These MBP-responsive T cells can be distinguished from host cells based on disparate expression of CD45 isoforms (CD45.1 defining the donor population) (17). The ability to track the pathogenic T cells provides a more refined system than previous studies and allows us to directly assess the impact of 1,25(OH)_2_D_3_ on autoreactive T cells. We administered 200 ng 1,25(OH)_2_D_3_ every 48 h from the day prior to immunization, until 7 days after immunization to ensure supplementation with 1,25(OH)_2_D_3_ during the majority of the priming phase of EAE. 1,25(OH)_2_D_3_ completely blocked the development of EAE, with none of the 14 mice developing disease, compared to 14 of the 15 vehicle-treated mice (Figure 1A). We analyzed four mice of each group at the peak of disease (on day 13) for the presence donor Tg4 T cells within CNS infiltrates and in the spleen. There was a significantly greater proportion of donor Tg4 cells among the CNS, but not the spleen, CD4+ T cells of the vehicle-treated mice (Figure 1B). Thus, 1,25(OH)_2_D_3_ administration impaired the induction of CNS autoimmune disease in this model, likely by limiting the access of MBP-reactive CD4+ T cells to the CNS.

The above finding of no shortage of Tg4 cells in the spleens of mice protected from EAE suggested that 1,25(OH)_2_D_3_ treatment might be able to suppress disease downstream of the activation of pathogenic potential in MBP-responsive T cells (e.g., by preventing their migration to the CNS). Modifying T cell migration to the CNS is at the root of two current therapeutics for MS (31, 32). We therefore asked whether 1,25(OH)_2_D_3_ treatment of host mice could prevent pathology driven by the infusion of pre-formed encephalitogenic Tg4 effector T cells. As shown in Figure 1C, by using previously described conditions for the *in vitro* activation and differentiation of Tg4 cells into pathogenic effectors for infusion into naïve mice (17), we found that the disease course was very similar in mice receiving 1,25(OH)_2_D_3_ or vehicle, with the majority of mice in both groups developing EAE. When assessed at day 18 post T cell transfer, there were no differences in the total numbers of CD4+ T cells, the numbers of donor Tg4 T cells, or the frequencies of donor Tg4 T cells within the CNS (Figure 1D and data not shown). The ability of 1,25(OH)_2_D_3_ to protect mice from EAE when actively induced by immunization, but not when passively transferred with effector T cells, therefore, indicated a primary impact on the initial stages of activation of potentially autoaggressive T cells.

### BMDC Generated in the Presence of 1,25(OH)_2_D_3_ Have a Reduced Ability to Prime Autoreactive T Cells *In Vitro*

**Figure 2:**
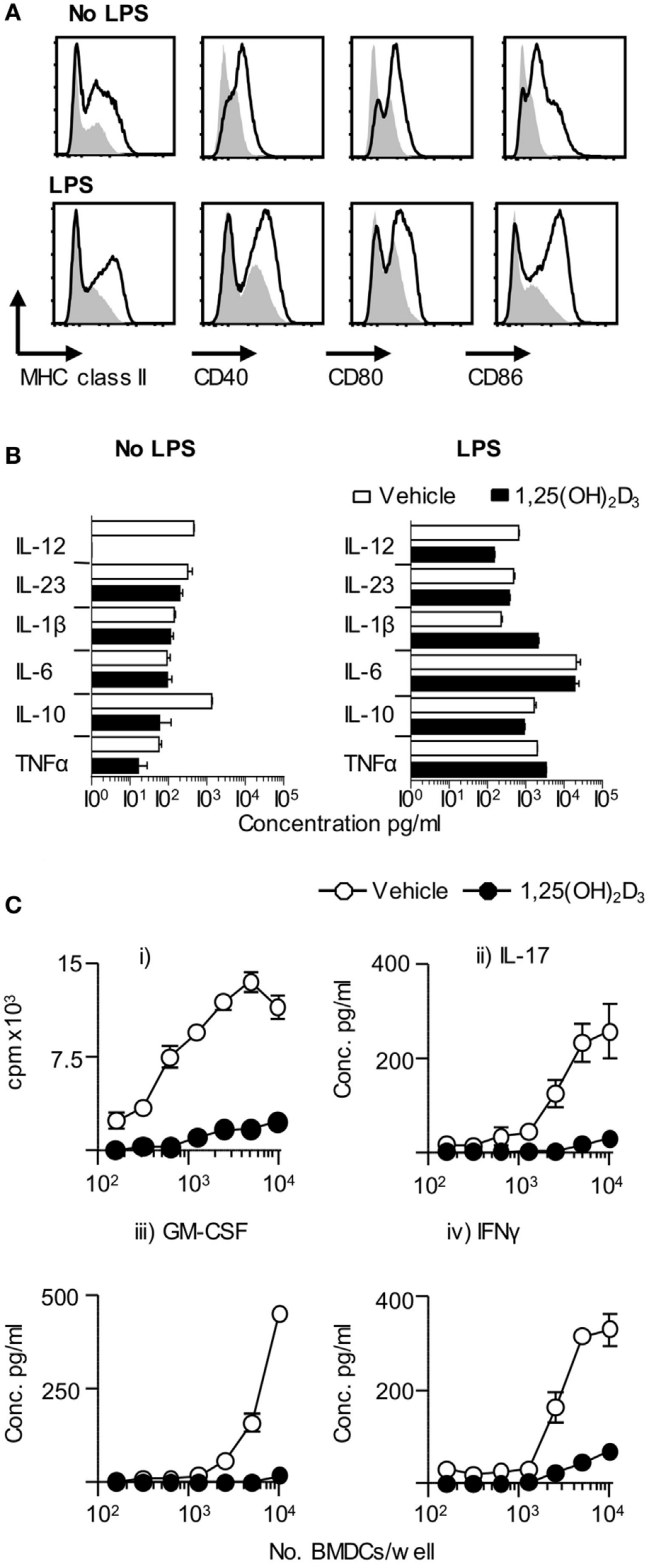
**VitD-BMDC have reduced ability to prime MBP reactive T cells *in vitro***. BMDC were generated and matured with LPS as described in Section “Materials and Methods.” **(A)** Expression of MHC class II, CD40, CD80, and CD86 on Veh-BMDC (black line) or VitD-BMDC (gray shade) following overnight maturation without or with LPS. **(B)** Cytokine levels in BMDC supernatants sampled after 18 h culture without and with LPS. **(C)** 2 × 10^4^ Tg4 CD4^+^ T cells were cultured with LPS-conditioned, MBP-pulsed BMDC and assessed for proliferation (thymidine incorporation). Concentrations of GM-CSF, IFN-γ, and IL-17 were measured in the supernatants after 72 h culture. Data are from one of four experiments giving consistent results.

Dendritic cells are key drivers of naïve T cell activation (1). In order to examine the effect of 1,25(OH)_2_D_3_ on DC function, we investigated the effects of restricting exposure to 1,25(OH)_2_D_3_ solely to those DC. Initial *in vitro* experiments showed that BMDC generated in the presence of 1,25(OH)_2_D_3_ (VitD-BMDC) had lower expression of MHC class II, CD40, CD80, and CD86 than vehicle treatment BMDC (Figure 2A). However, the addition of 1,25(OH)_2_D_3_ to BMDC cultures did not restrict the capacity of BMDC to produce EAE-associated innate cytokines in response to LPS stimulation (Figure 2B). Furthermore, there was no significant increase in IL-10 production by BMDC exposed to 1,25(OH)_2_D following LPS maturation (*p* = 0.11, Student’s *t*-test). Nevertheless, these BMDC did show an impaired ability to trigger the clonal expansion of naïve Tg4 T cells and to promote their production of pro-inflammatory effector cytokines, such as IL-17A, IFN-γ, and GM-CSF *in vitro* (Figure 2C). IL-10 was not detectable in the supernatants of either Veh-BMDC or Vit-BMDC and Tg4 co-cultures (data not shown).

### 1,25(OH)_2_D_3_-Conditioned BMDC Have a Reduced Ability to Initiate EAE

**Figure 3:**
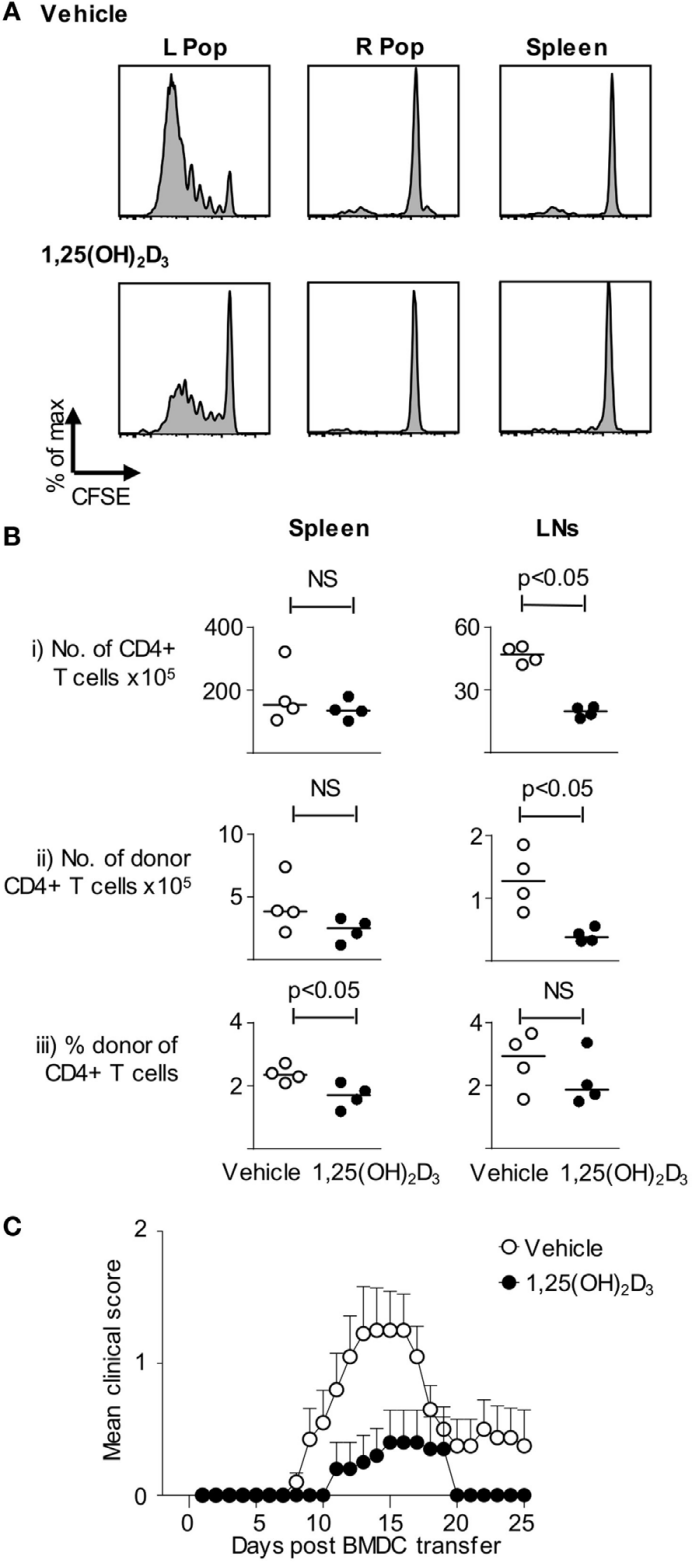
**VitD-BMDC have reduced ability to initiate EAE**. **(A)** B10.PL received CFSE-loaded 2 × 10^6^ Tg4.CD45.1 CD4**+** T cells 1 day prior to injection of 2 × 10^4^ MBP-loaded Veh-BMDC or VitD-BMDC into the left hindleg. The spleen and right and left popliteal lymph nodes (PLN) were harvested separately and single-cell preparations were made. Plots show CFSE-dilution gated on CD45.1+ T cells from the indicated organs. Results shown are representative of two independent experiments. **(B)** B10.PLxC57BL/6 mice received 2.5 × 10^6^ Tg4.CD45.1 CD4+ T cells 1 day prior to injection of 2 × 10^6^ MBP-loaded BMDC. Six days later, lymphoid organs were harvested for FACS analysis of total CD4+ and donor CD45.1+ cells. Cell numbers and percentages were compared by a Mann–Whitney *U* test. **(C)** 2 × 10^6^ Tg4.CD45.1 T cells were transferred into B10.PLxC57BL/6 mice 1 day prior to injection of 2 × 10^6^ LPS-conditioned, MBP-loaded Veh-BMDC or VitD-BMDC sc in each hindlimb. Pertussis toxin was administered i.p at time of BMDC transfer and 2 days later. Results shown are pooled from three independent experiments.

To extend the above *in vitro* observations to the *in vivo* setting, host mice were seeded with CFSE-labeled naive Tg4 T cells and then received BMDC pulsed with Ac1-9(4Tyr) MBP generated in presence of either vehicle or 1,25(OH)_2_D_3_. Sampling the mice 3 days after DC injection revealed that the Tg4 cells proliferated (as assessed by CFSE-dilution) only in the draining lymph node and not in the contralateral lymph node or the spleen. This proliferation was markedly less in mice receiving VitD-BMDC than in those receiving vehicle-treated DC (Figure 3A). Similar experiments using naïve Tg4 T cells that were not loaded with CFSE, and sampling lymphoid organs 6 days after DC transfer, revealed differences in the lymph nodes that drained the site of DC injection. Donor Tg4 cells showed evidence of TCR interaction with their cognate peptide–MHC complex (a CD62L^lo^CD44^hi^ phenotype) regardless of whether or not the DC were exposed to 1,25(OH)_2_D_3_. However, the numbers of donor CD4+ Tg4 cells present in the draining lymph nodes were lower in mice receiving the VitD-BMDC (Figure 3B).

Having determined that 1,25(OH)_2_D_3_-exposed DC were less effective at priming Tg4 cells *in vitro* and *in vivo*, our next step was to confirm the consequence of this on CNS pathology. As shown in Figure 3C, the incidence of EAE was significantly higher in those mice receiving vehicle-conditioned BMDC (14/20) compared to those mice receiving VitD-BMDC (3/20) (Fischer’s exact test, *p* < 0.001). The maximum EAE score was also significantly higher in mice receiving Veh-BMDC (median score Veh-BMDC 1.5, VitD-BMDC 0. Mann–Whitney *U* test, *p* < 0.005).

### 1,25(OH)_2_D_3_-Conditioned BMDC Do Not Tolerize MBP-Reactive T Cells

**Figure 4:**
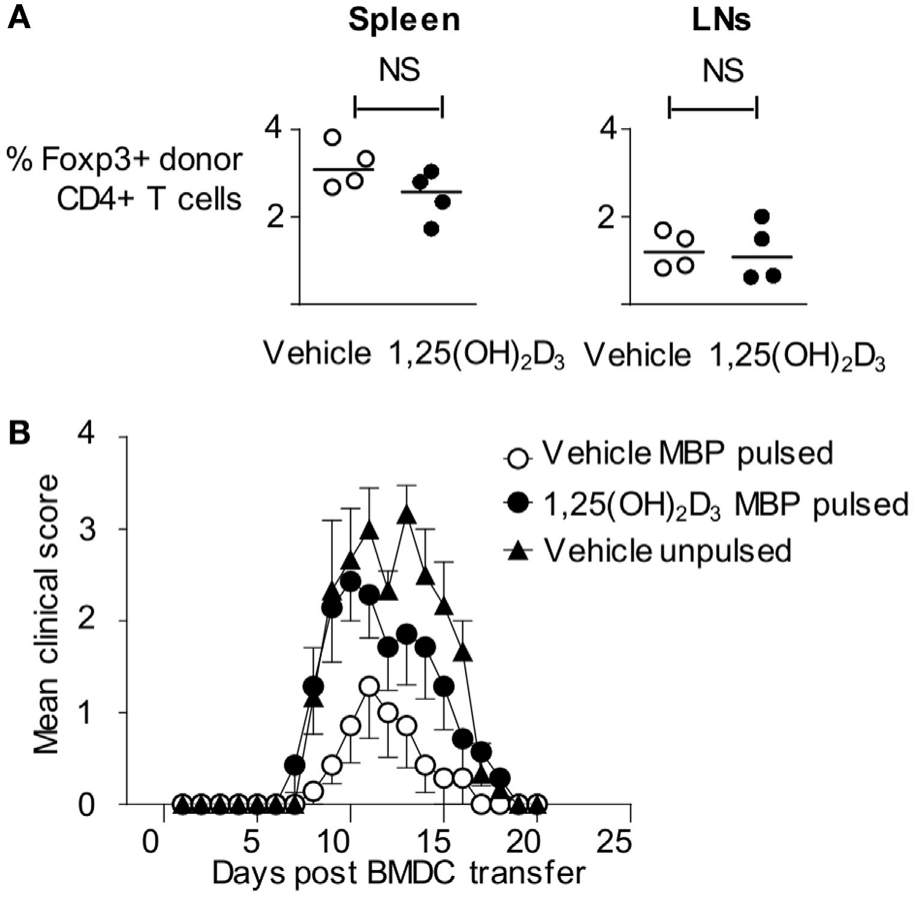
**Failure of VitD-BMDC to initiate EAE is not due to a tolerizing effect on T cells**. **(A)** B10.PLxC57BL/6 mice were administered 2.5 × 10^6^ CD4 + CD45.1 Tg4 T cells i.v followed by 2 × 10^6^ LPS-conditioned, MBP-loaded BMDCs s/c in the hindlimb on the following day. The draining lymph nodes and spleen were harvested at day 6 and single-cell preparations were made. Percentage of Foxp3+ cells in donor CD45.1+ T cells from spleen and draining lymph nodes are shown. **(B)** 2 × 10^6^ CD4 + CD45.1 Tg4 T cells were transferred into B10.PLxC57BL/6 recipients. On the following day, 2 × 10^6^ Veh-BMDC or VitD-BMDC were injected s/c in hindlimb. Mice were administered 10 μg Ac1-9(4Tyr) MBP and CFA 6 days later. Pertussis toxin was administered i.p on day of CFA administration and 2 days later. The clinical disease course is shown.

As described above, Tg4 cells showed evidence of TCR ligation *in vivo* in response to 1,25(OH)_2_D_3_-conditioned BMDC. This leads to significantly reduced pathogenic activity in the Tg4 T cells and, instead, might have rendered them unresponsive to subsequent activation (i.e., the VitD-BMDC might induce immunological tolerance to MBP Ac1-9). This possibility was initially investigated by examining the expression of Foxp3, a transcription factor expressed in regulatory T cells (33), expression in host and donor Tg4 cells 6 days following BMDC transfer. There was no significant difference in the proportion of donor Tg4 cells expressing Foxp3, indicating that VitD-BMDC did not induce a regulatory population of Tg4 cells *in vivo* (Figure 4A). We next investigated whether VitD-BMDC could tolerize T cells through other, non-Foxp3 mediated, mechanism. We used the above Tg4 then DC transfer protocol and, 7 days after DC injection, immunized the mice for EAE induction with MBP peptide in CFA. The incidence of EAE was equivalent between groups of mice receiving vehicle-treated DC (4/7) or 1,25(OH)_2_D_3_-conditioned DC (6/7). We, therefore, found no evidence to support the induction of long-term Ag-specific unresponsiveness in this model by the use of VitD-BMDC (Figure 4B).

### Addition of 1,25(OH)_2_D_3_ to BMDC Cultures Results in a Lower Proportion of CD11c+ Cells

**Figure 5:**
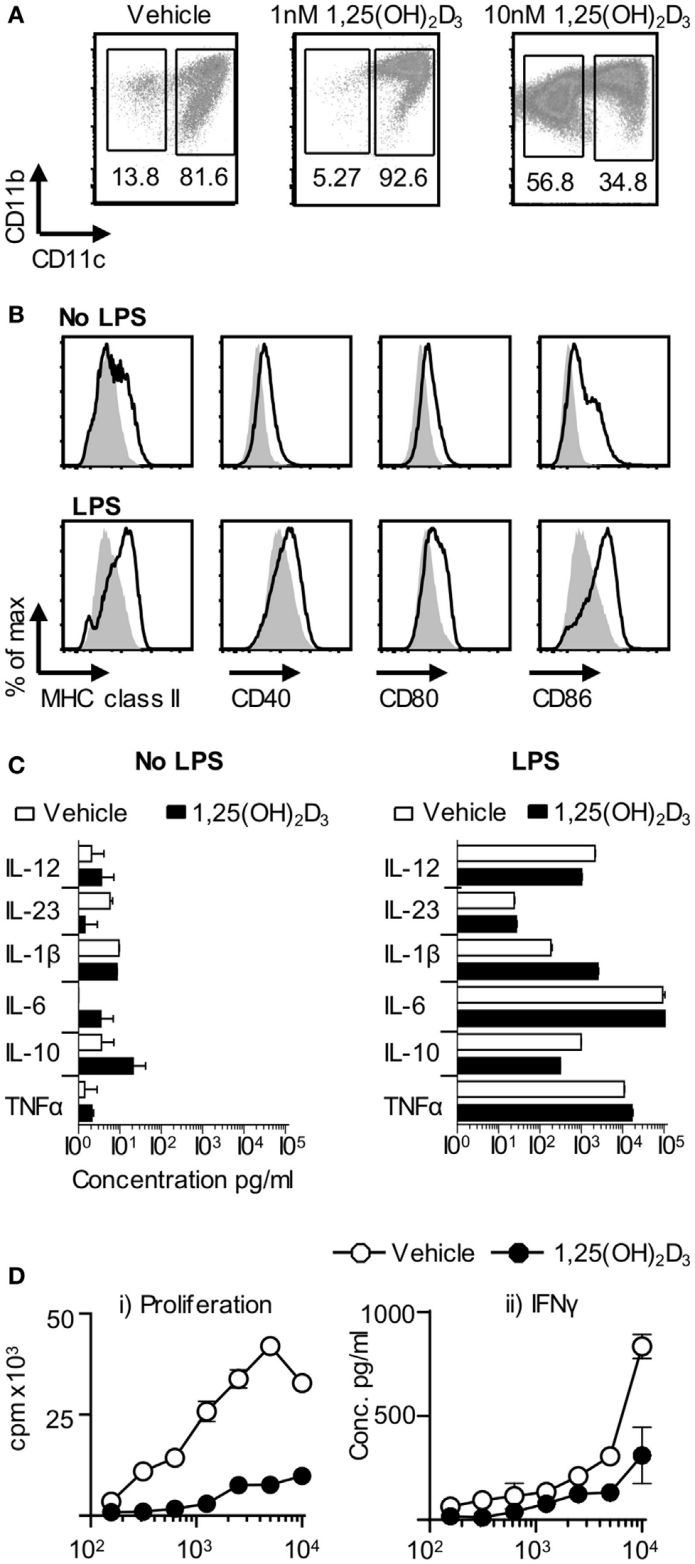
**VitD-BMDC have a lower proportion of CD11c**+** cells**. **(A)** BMDCs were generated in presence of 0, 1, or 10 nM of 1,25(OH)_2_D for 9 days. CD11b and CD11c expression was assessed by flow cytometry. **(B)** Expression of MHC class II, CD40, CD80, and CD86 on Veh-BMDC (black line) or VitD-BMDC (gray shade) gated on CD11c+ cells following overnight maturation without or with LPS. **(C)** Cytokine production by CD11c+ Veh-BMDC (open bar) and CD11c+ VitD-BMDC (black bar) without or with overnight LPS maturation. **(D)** 2 × 10^4^ Tg4 CD4+ T cells were cultured with LPS-matured Veh-BMDC (open circle) or VitD-BMDC (black circle) for 72 h and assessed for proliferation and production of IFNγ. Data are from one of four experiments giving consistent results.

In light of the failure of VitD-BMDC to tolerize naïve Tg4 T cells *in vivo*, we next investigated whether the inability of VitD-BMDC to robustly induce EAE was due to an altered phenotype of the BMDC, which were generated in the presence of 1,25(OH)_2_D_3_. We performed flow cytometry analysis on the BMDC at day 9 and found that the addition of 1,25(OH)_2_D_3_ to the BMDC cultures resulted in a dose-dependent decrease in the proportion of CD11c+ cells (Figure 5A). Analysis of the CD11c negative cells demonstrated that they were all MHC class II negative. The expression of MHC class II, CD40, CD80, and CD86 was also significantly lower on CD11c+ cells before and after LPS stimulation (Figure 5B). The CD11c+ cells from vehicle and VitD-BMDC cultures were isolated by FACS sorting and cytokine production measured following overnight stimulation with LPS. Production of cytokines involved in activating T cells was similar from both CD11c+ and BMDC populations (Figure 5C). Next, we examined whether there was a differential ability of vehicle or CD11c+ VitD-BMDC to prime Tg4 CD4+ T cells. We discovered that CD11c+ VitD-BMDC were much less effective at inducing proliferation and effector cytokine production from naïve T cells (Figure 5D).

### VitD-CD11c+ BMDC are Effective at Priming T Cells *In Vivo* and at Initiating EAE

**Figure 6:**
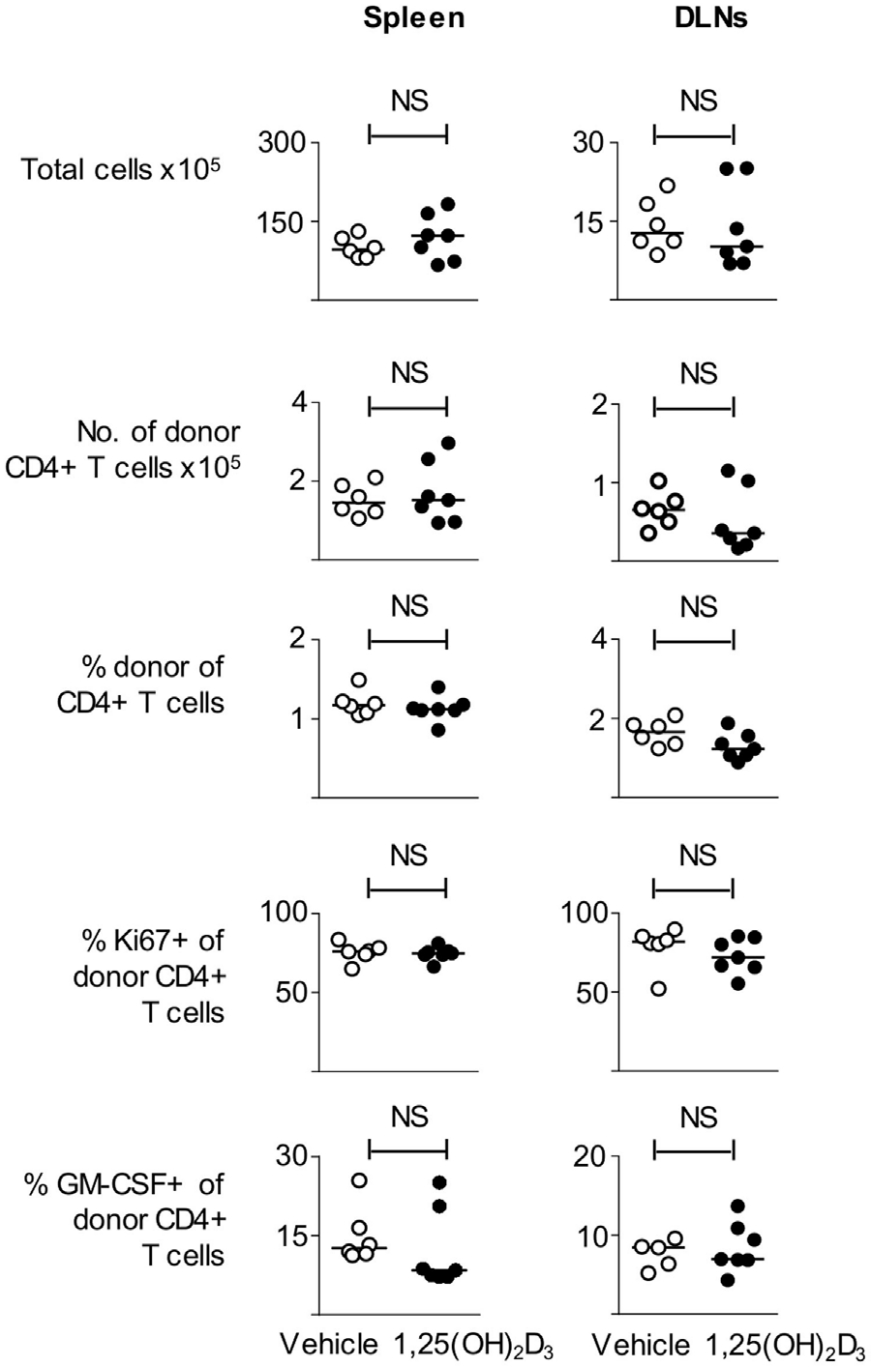
**VitD-CD11c **+** BMDC can effectively prime T cells *in vivo***. B10.PLxC57BL/6 mice received 2 **×** 10^6^ Tg4.CD45.1 CD4^+^ T cells 1 day prior to s/c injection of 1 × 10^5^ MBP-loaded CD11c+ Veh-BMDC or CD11c+ VitD-BMDC into the hindlimb. Six days later, spleen and draining lymph nodes were harvested for FACS analysis of total CD4^+^ and donor CD45.1^+^ cells. The total numbers of cells, number of donor Tg4 cells, percentage of donor Tg4 cells of total CD4+ cells, percentage of Ki67+ of donor Tg4 cells, and percentage of GM-CSF+ donor Tg4 are shown in spleen and DLN. Data are from one of two experiments giving consistent results.

Next, we examined whether CD11c+ VitD-BMDC were as effective as CD11c+ Veh-BMDC at priming T cells *in vivo*. We immunized host mice seeded with Tg4 CD4+ T cells with either FACS sorted CD11c+ Veh-BMDC or CD11c+ VitD-BMDC and harvested spleen and draining lymph nodes 6 days later. We found that there was no difference in the number of total or donor Tg4 CD4+ T cells or percentage of donor Tg4 cells of total CD4+ T cells (Figure 6). There was also no statistical difference in the proportion of donor Tg4 CD4+ T cells that were Ki67+ or in the proportion of cells that were GM-CSF+ following overnight stimulation with MBP (Figure 6).

**Figure 7:**
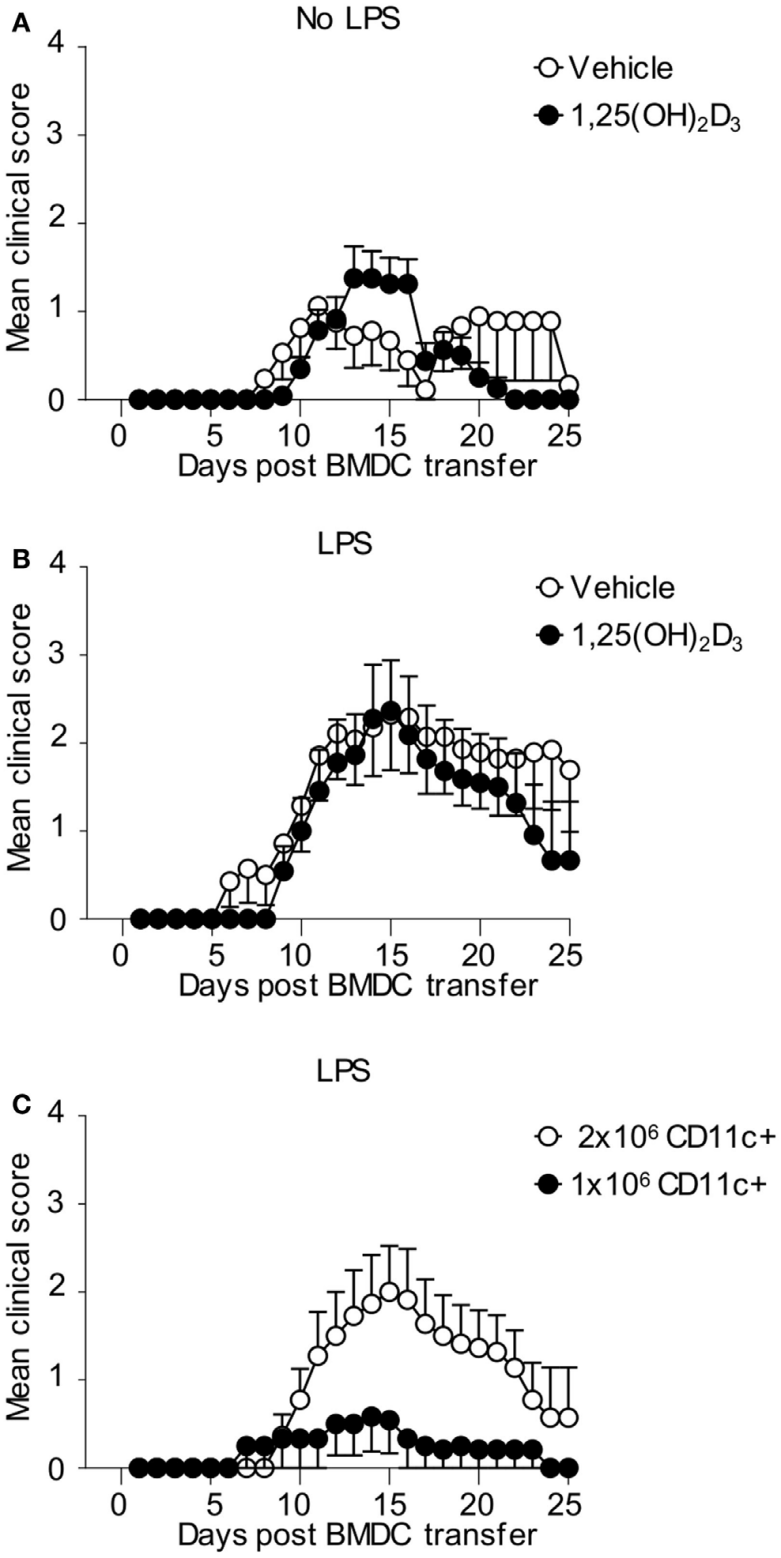
**CD11c**+** VitD-BMDC are highly effective at initiating EAE**. 1 × 10^6^ CD4 + CD45.1 Tg4 T cells were transferred into B10.PLxC57BL/6 recipients. One day later, 2 × 10^6^ MBP-pulsed CD11c+ Veh-BMDC or VitD-BMDC, which had been matured overnight either without LPS **(A)** (Veh *n* = 17, VitD *n* = 22) or LPS **(B)** (Veh *n* = 14, *n* = 11), were injected s/c in hindlimb. Pertussis toxin was administered i.p at days 0 and 2 post CD11c+ BMDC transfer. The clinical disease course is shown. Disease course is shown from three pooled experiments. **(C)** 1 × 10^6^ CD4 + CD45.1 Tg4 T cells were transferred into B10.PLxC57BL/6 recipients. One day later, 2 × 10^6^ (*n* = 11) or 1 × 10^6^ (*n* = 12) MBP-pulsed, LPS-matured CD11c+ VitD-BMDC were injected s/c in hindlimb. Pertussis toxin was administered i.p at days 0 and 2 post CD11c+ BMDC transfer. The clinical disease course is shown. Disease course is shown from three pooled experiments.

Finally, we examined whether CD11c+ VitD-BMDC were as effective at inducing EAE as CD11c+ Veh-BMDC on a per cell basis. We immunized host mice seeded with Tg4 CD4+ T cells with either 2 × 10^6^ FACS sorted CD11c+ Veh-BMDC or VitD-BMDC that had been cultured overnight with LPS or without LPS. CD11c+ Vit-BMDC that were not LPS matured were just as effective at inducing EAE as non-LPS matured, CD11c+ Veh-BMDC (Figure 7A). To examine the role of LPS further, we next repeated the experiments but matured the FACS sorted CD11c+ BMDC with LPS overnight prior to transfer. We found that LPS-conditioned CD11c+ VitD-BMDC were again similarly pathogenic as LPS-conditioned CD11c+ Veh-BMDC (Figure 7B). These data demonstrate that on a per cell basis the addition of 1,25(OH)_2_D_3_ has no effect on restraining the ability of CD11c+ to induce a pathogenic T cell response *in vivo*. Therefore, to examine whether the failure of the unsorted VitD-BMDC to induce EAE was simply down to the lower proportion of CD11c+ cells, we next immunized host mice with either 1 × 10^6^ or 2 × 10^6^ LPS-matured CD11c+ VitD-BMDC. This number of cells was used as approximately half of the VitD-BMDC were CD11c+ (Figure 5A). Only 2 of 12 mice immunized with 1 million CD11c+ VitD-BMDC developed EAE compared to 7 of 11 mice immunized with 2 million CD11c+ VitD-BMDC (Fisher’s exact test, *p* = 0.036). Furthermore, the median peak disease score was also significantly lower (Mann–Whitney *U* test, median Veh-BMDC 2.5, median VitD-BMDC 0, *p* = 0.036) (Figure 7C).

## Discussion

The administration of vitamin D has beneficial properties in a range of experimental immunopathological conditions (20–30, 34) and its clinical translation is currently being evaluated in MS (35). Our study revealed that 1,25(OH)_2_D_3_ could suppress EAE driven by active immunization with MBP autoantigen in CFA, but could not suppress the development of passive EAE following transfer of pre-formed MBP-responsive effector cells. This implied an immunomodulatory effect of 1,25(OH)_2_D_3_ that was evident early in the activation of the T cells, before they had reached the point of being fully differentiated effector T cells. Since DC are key initiators of naïve T cell activation *in vivo*, we examined the effects of 1,25(OH)_2_D_3_ on DC phenotype and function. We found that that VitD-BMDC had lower expression of MHC class II, CD80, and CD86, which is consistent with other studies of murine and human DC (5, 34, 36–39). By contrast, we found no evidence that 1,25(OH)_2_D_3_-conditioning could significantly alter the ability of BMDC to produce pro-inflammatory cytokines in response to LPS. Although the production of EAE-associated cytokines by DC remains intact in VitD-BMDC, there was a failure to induce T cell proliferation both *in vitro* and *in vivo*. Importantly, our novel BMDC-driven EAE model allowed us to probe the effects of VitD-BMDC on T cells that have the potential to initiate autoimmune pathology *in vivo* (15). We found that VitD-BMDC were rarely able to drive an autopathogenic T cell response with only 15% of mice developing EAE compared to 70% of mice immunized with Veh-BMDC.

Arguably, the most plausible explanation for the failure of VitD-BMDC to robustly induce EAE would be the induction of T cells with a suppressive phenotype (9, 34, 36, 40, 41), which may be associated with enhanced production of IL-10 by BMDC conditioned with 1,25(OH)_2_D_3_ (36, 38). We found no evidence for increased DC IL-10 production, or a switch to a regulatory T cell phenotype. Furthermore, T cells exposed to VitD-BMDC *in vitro* did not produce elevated levels of IL-10, we found no increase in Foxp3 expression by transferred Tg4 T cells *in vivo* and, perhaps most tellingly, VitD-BMDC transfer did not render mice resistant to the subsequent induction of EAE by immunization with the MBP peptide in CFA. This latter observation came as something of a surprise, since the induction of antigen-specific tolerance in T cells exposed *in vivo* to weak signals 1 and 2 is a basic immunological paradigm (42). It remains possible that the ability of the transferred DC to produce innate cytokines prevents the establishment of the tolerogenic signaling program within the Tg4 T cells, leaving them capable of fully pathogenic function upon subsequent strong activation, as we found. Alternatively, the subcutaneous administration of the DC may well have been insufficient to provide a global tolerogenic stimulus to all Tg4 T cells within the host mice, as the reduced cell proliferation in the draining lymph node after transfer of BMDC would indicate. Further studies are required to examine whether 1,25(OH)_2_D has different effects on the subsets of CD11c+MHCclassII + high BMDCs which have been recently discovered (43).

To examine whether impaired ability to prime T cells was the cause of the failure of VitD-BMDC to induce robust EAE, we performed further phenotyping on VitD-BMDC by flow cytometry. We found that the addition of 1,25(OH)_2_D_3_ to BMDC cultures resulted in a reduction of CD11c+ cells, a finding that is consistent with a previous study (44). The CD11c^−^ cells were MHC class II negative and were unable to prime T cells *in vitro*. We discovered that the CD11c+ VitD-BMDC were significantly less effective at priming T cells *in vitro* yet were just as effective at initiating EAE as CD11c+ Veh-BMDC. When the mice were immunized with similar numbers of CD11c+ cells as were present in initial VitD-BMDC transfers, there was the same failure to robustly initiate EAE. This finding demonstrates that the inability of VitD-BMDC to robustly initiate EAE is due to the lower number of CD11c+ cells present in the VitD-BMDC cultures.

Our data raise important concerns to the developing field of DC immunotherapy. In light of the effects of 1,25(OH)_2_D_3_ on DC phenotype *in vitro* and *in vivo*, 1,25(OH)_2_D_3_ has been widely used to generate tolerogenic DC (2). Tolerogenic DCs have been used to prevent and ameliorate autoimmune pathology in a wide range of experimental murine autoimmune systems, including collagen-induced arthritis, non-obese diabetic mice, and EAE models (34, 45, 46). The success of the tolerogenic DC treatments in experimental systems has resulted in their translation to therapies for humans with autoimmune diseases (2). For example, a phase 1 trial of tolerogenic DC treatment in patients with type 1 diabetes has been undertaken (47) and a trial in rheumatoid arthritis using 1,25(OH)_2_D_3_-conditioned tolerogenic DC is ongoing (11). 1,25(OH)_2_D_3_-conditioned monocyte-derived DCs from MS patients have been shown to induce hyporesponsiveness in myelin-responsive T cells *in vitro*, a finding that has fueled interest in the development of tolerogenic DC therapies for MS (48). However, it is important to acknowledge that the approaches used to generate many of the tolerogenic DC which have been used clinically are not exactly the same as the BMDC generation methodology used in this study.

Although tolerogenic DCs appear to be a promising immunotherapy, the administration of autoantigen-loaded DC is not without risk of side effects, particularly regarding their potential to further activate autoreactive T cells. The safety of tolerogenic DC has been difficult to assess since there have been few studies that have robustly demonstrated that a transfer of *ex vivo* generated DC can initiate autoimmunity. Although previous studies have attempted to use BMDC to induce EAE, no such model has been adopted widely (12–14). This has resulted in the impression that the transfer of *ex vivo*-generated DC is unlikely to generate an autopathogenic T cell response.

Our recently described BMDC-induced EAE model has allowed us, for the first time, to examine the effects of 1,25(OH)_2_D_3_ on BMDC function in a model where BMDC can robustly induce EAE. Our results demonstrate that conditions that have been reported to induce tolerogenic DC in some settings do not always result in the generation of DC that can tolerize T cells *in vivo* (2). Indeed, we have shown that the apparent failure of VitD-BMDC to initiate autoimmune pathology is due to the lower number of CD11c+ cells that emerge in BMDC cultures when 1,25(OH)_2_D_3_ is added. Our results caution against the assumption that 1,25(OH)_2_D_3_ invariably induces a tolerogenic phenotype in BMDC even when they phenotypically and functionally show typical features of tolerogenic DC such as lower expression of co-stimulatory molecules and reduced ability to prime T cells *in vitro* (2). Our study also highlights the difficulties in predicting *in vivo* BMDC function based on *in vitro* analysis. The CD11c+ VitD-BMDC were significantly less effective at initiating proliferation and pro-inflammatory cytokine production from naïve T cells *in vitro* yet were equally effective as CD11c+ Veh-BMDC at initiating clinical CNS autoimmune pathology *in vivo*. The addition of the TLR-4 agonist LPS, which has been used to generate tolerogenic DC (11, 49), did not influence this result as CD11c+ VitD-BMDC were equally effective as vehicle-treated counterparts regardless of whether they were matured with or without LPS.

Our finding that 1,25(OH)_2_D_3_ can ameliorate active but not passive EAE is of interest when considering the possible beneficial use of 1,25(OH)_2_D_3_ in relapsing-remitting MS. First, by analogy with the inability of 1,25(OH)_2_D_3_ to modulate passive EAE, it seems unlikely that 1,25(OH)_2_D_3_ would affect the autoimmune (T cell) component of a relapse, in which fully activated effectors would be expected to be at play. Second, if a relapse provoked by reactivation of myelin-responsive memory T cells is dependent on presentation of the autoantigen by DC or other antigen-presenting innate immune cells, then 1,25(OH)_2_D_3_ might be able to limit this. However, our study demonstrates that if 1,25(OH)_2_D_3_ administration impairs the ability of BMDC to drive pro-inflammatory cytokines from T cells, this may not be sufficient to limit CNS autoimmune pathology. Third, given the unpredictable timing of MS relapses, and our observation that pathogenic activity was not irretrievably lost by the T cells that remained in mice receiving 1,25(OH)_2_D_3_-conditioned DC, the prediction might be that long-term use of 1,25(OH)_2_D_3_ administration would be the option most-likely to succeed.

In summary, our data demonstrate that VitD-BMDC have a greatly reduced ability to initiate EAE. However, this was not due to their ability to induce tolerance in autoreactive T cells. Rather, it was due to the reduced numbers of CD11c+ cells that were present in VitD-BMDC populations since sorted populations of CD11c+ VitD-BMDC were equally as effective at initiating EAE. Our study highlights the difficulties of predicting *in vivo* DC function based on *in vitro* assays and demonstrates the potent ability of CD11c+ VitD-BMDC to drive an autopathogenic T cell response.

## Author Contributions

Conceived and designed the experiments: DB, RM, SA, and AM. Performed the experiments: DB, LS, RO, ML, and RM. Analyzed the data: DB, LS, RM, and SA. Wrote the paper: DB, LS, RO, ML, AM, SA, and RM.

## Conflict of Interest Statement

The authors declare that the research was conducted in the absence of any commercial or financial relationships that could be construed as a potential conflict of interest.
